# EZH2 inhibition activates dsRNA-interferon axis stress and promotes response to PD-1 checkpoint blockade in NSCLC

**DOI:** 10.7150/jca.73291

**Published:** 2022-07-04

**Authors:** Fengqi Qiu, Qi Yang, Wenjia Sun, Kexin Ruan, Nan Jiang, Jianya Zhou

**Affiliations:** Department of Respiratory Disease, Thoracic Disease Center, The First Affiliated Hospital, School of Medicine, Zhejiang University, Hangzhou, China.

**Keywords:** Epigenetic regulation, Immune therapy, MDA5, Non-small cell lung cancer.

## Abstract

Lung cancer is the leading cause of cancer death and immunotherapy had been approved be a useful approach for NSCLC therapy. However, only part of the patients responds to checkpoint inhibitors. The EZH2, as a histone modification regulator, is overexpressed in NSCLC and negatively regulates the interferon-stimulated genes. Here, we demonstrate that EZH2 inhibition increases the double-strand RNA (dsRNA) level and then triggers the IFN pathway stress which is dependent on the pattern recognition receptors (TLR3, MDA5). The antigen presentation genes and PDL1 were also upregulated by inhibition of EZH2. Furthermore, in the immunocompetent LLC tumor model, the inhibition of EZH2 causes tumor regression and enhances the CD8^+^T cell infiltration. The EZH2 depletion triggers significant responses of the LLC mouse model to anti-PD1 therapy. This study identifies that inhibition of EZH2 promotes the dsRNA interferon driven antitumor immunity and enhances the anti-PD1 antitumor efficacy in NSCLC. These data suggest that EZH2 inhibition combined with anti-PD1/PDL1 is a promising lung cancer treatment strategy.

## Introduction

Lung cancer is the most commonly diagnosed malignancy and the leading cause of cancer death globally 1. Immune checkpoint inhibitors targeting PD-1/PD-L1 have become the standard first-line treatment for advanced non-small cell lung cancer (NSCLC) patients 2. However, there was still a large proportion of patients who cannot benefit from current targeted therapies. In an unselected previously treated NSCLC population, response rates with single-agent immune checkpoint inhibitors (ICIs) varied from 14% to 20%, as well as 15% to 25% in chemotherapy-refractory patients 3-5. Resistance toward antibody blocking PD1/PDL1 could be explained by low tumor immunogenicity and an immunosuppressive tumor microenvironment including lack of T cell infiltration and dysfunctional T cells, as well as recognition insufficient by T cells 6.

EZH2 is a core component of polycomb repressor complex 2 (PRC2) which catalyzes trimethylation on histone 3 lysine 27 (H3K27me3) and represses gene expression 7. EZH2 is overexpressed in various tumors, such as breast cancer, colorectal cancer, and prostate cancer, and contributor to cancer initiation and progression 8, 9. Despite its crucial role in tumors, EZH2 has attracted attention for its function in enhancing inflammatory response 10. However, the impact of EZH2 on the tumor immune microenvironments and the efficacy of immunotherapy is not fully understood in NSCLC patients.

Growing evidence showed that interferons (IFNs) are major regulators of tumor-infiltrating immune cells and induce effective immune response 11. In addition, previous studies demonstrated that the double-strand RNA (dsRNA) derived from endogenous retroviruses (ERVs) could trigger interferon activation, and further, lead to alteration in the local microenvironment and boost response to immune checkpoint therapy 12. Several epigenetic modifiers including DNMT1 and LSD1 had been revealed to prevent dsRNA expression by restraining ERVs levels in melanoma 13. A recent study revealed that EZH2 inhibition activates dsRNA and potentiates prostate cancer response to PD-1 checkpoint blockade 14. However, it remains unclear whether EHZ2 may be involved in the dsRNA expression and IFNs activation in non-small cell lung cancer.

Epigenetic regulation mediated by EZH2 has not been fully studied for its ability to induce a response to anti-PD1 in NSCLC. In this study, either by genetic knockdown or by pharmacologic inhibition, we demonstrate that targeting EZH2 depresses endogenous dsRNA or causes IFNs stimulation. In the tumor model, EZH2 inhibition triggers antigen processing and promotes anti-tumor T cell infiltration, and potentiates anti-PD1 therapy. The present study indicates that targeting EZH2 in combination with anti-PD1/PDL1 may provide a new treatment strategy for NSCLC. In general, these studies provide evidence for targeting the epigenetic regulator of EZH2 to enhance the immunotherapy effect in NSCLC.

## Methods and Materials

### Cell lines and treatment

Human lung adenocarcinoma cell line (A549, SK-MES1) and squamous carcinoma cell line (H1299, H520), as well as Mouse LLC(Lewis Lung Carcinoma Cell), were purchased from American Type Culture Collection (ATCC). All cell lines were maintained in Dulbecco's modified Eagle's medium (DMEM) supplemented with 10% fetal bovine serum (FBS) and 1% penicillin-streptomycin. All cell lines were cultured in a 37°C incubator with 5% CO_2_, and sub-cultured every 2-3 days. The highly selective EZH2 inhibitor, GSK126, was purchased from Selleck Chemicals. The cells were seeded in 6-well or 12-well plates, 24 hours later, and were treated with 2uM GSK126, DMSO, or IFN-γ (100 ng/ml) for 6 days. During the treatment, cells were passaged once and refilled with fresh GSK126 or indicated control regents.

### Flow cytometry

*In vitro* analysis of dsRNA. The cells were treated for indicated days. For dsRNA staining, the cells were digested to single-cell suspension with recombinant trypsin EDTA Solution (HAKATA), then the trypsin solution was deactivated in DMEM (BasalMedia) suspension with 10% FBS (GeminiBio). Cells were washed with PBS and then fixed with 4% paraformaldehyde for 10 to 20 min at room temperature. Centrifuged the cells at 1000g for 5min and then washed once with PBS. The cells were resuspended with 100ul PBS and permeabilized with the ice-cold methanol at a final concentration of 90% methanol. The cells were incubated in methanol for 30 min on ice and then washed twice with MACS buffer (PBS supplemented with 2% FBS and 2 mM EDTA). The cells were incubated in primary dsRNA antibody (J2, Scicons) at a concentration of 1:200 overnight in the dark at 4°C. The cells were washed twice with MACS and incubated with Alexa Fluor™ 488 conjugated goat anti-mouse antibody for 1 hour followed by twice washing with MACS buffer. Samples were tested using BD LSR Fortessa (BD Biosciences) and subsequently analyzed using FlowJo software (Treestar).

*In vivo* tumor analysis. The mouse was anesthetized and sacrificed on day14 post tumor implantation. The tumors were isolated and cut into 2mm sized pieces incubate in collagenase and DNase for 30 minutes at 37°C. Samples were dissociated into single cell suspension and passed through a 70 um filter. For tumor staining, the samples were stained with MCH-1 (PE anti-mouse H-2Kb Antibody, Biolegend), as well as CD45.2 (Pacific Blue™ anti-mouse CD45.2 Antibody, Biolegend). 7-AAD was used for dead cell discrimination before applying it to flow cytometry. For infiltrating leukocyte staining, the cell suspension was spun through a percoll gradient to enrich leukocytes. Cells were washed twice and stained with antibodies list below: CD45.2-Pacific Blue (Biolegend 109819), CD3e-Brilliant Violet 510™ (Biolegend 100233), CD4-Alexa Fluor® 700, (Biolegend 100536), CD8a-Brilliant Violet 605™ (Biolegend 100743), CD11b-FITC, (Biolegend 101205), Gr-1-APC-CY7 (Biolegend 108423). Samples were tested using BD LSR Fortessa (BD Biosciences) and subsequently analyzed using FlowJo software.

### dsRNA Immunofluorescent staining

The dsRNA immunofluorescent staining steps were performed as previously described with modification 15, 16. Briefly, adherent cells were washed with PBS and fixed with 4% paraformaldehyde at room temperature for 15min. Cells were washed with PBS and then blocked with 2% BSA, 0.1 Triton-X100 in PBS. The samples were incubated with dsRNA(J2, Scicons) primary antibody overnight at 4°C. Then the second antibody AF488 conjugated goat anti-mouse antibody was used to incubate for 1 hour. The DAPI Staining Solution (Abcam) was used for DNA counterstaining. Zeiss LSM 510 Meta Inverted Confocal Microscope was used to take the images.

### RT-qPCR

For total RNA extraction, cells were lysed in RNAiso plus (Takara, cat#9108) prior to RNA extraction. The RNA was harvested according to the manufacturer's protocol. The extracted RNA was transcribed into cDNA immediately using the PrimeScript RT Master Mix Kit (Takara, cat#RR036a) according to the manufacturer's instruction. The cDNA was ready for real-time quantitative PCR. The NovoStart SYBR qPCR SuperMix Plus (Novoprotein, cat#E096-01A) was used for PCR amplification with the manufacturer's recommended conditions. Real-time amplification cycles' data were collected by LightCycler 480 system (Roche). Data were normalized to the GAPDH to obtain the ΔCT values that were used to calculate the fold change from the ΔΔCT values following normalization to the control group. The primers used are listed below: human IFN-α: F-GCCTCGCCCTTTGCTTTACT, R-CTGTGGGTCTCAGGGAGATCA; human IFN-β: F-ATGACCAACAAGTGTCTCCTCC, R-GGAATCCAAGCAAGTTGTAGCTC; human IL-28b: F-TAAGAGGGCCAAAGATGCCTT, R-CTGGTCCAAGACATCCCCC; human OASL: F-CTGATGCAGGAACTGTATAGCAC, R-CACAGCGTCTAGCACCTCTT; human ISG15: F-CGCAGATCACCCAGAAGATCG, R-TTCGTCGCATTTGTCCACCA; human TLR3: F-TTGCCTTGTATCTACTTTTGGGG, R-TCAACACTGTTATGTTTGTGGGT; human MDA5: F-TCGAATGGGTATTCCACAGACG, R-GTGGCGACTGTCCTCTGAA; human RIG-I: F-CTGGACCCTACCTACATCCTG, R-GGCATCCAAAAAGCCACGG; human GAPDH: F-GGAGCGAGATCCCTCCAAAAT, R-GGCTGTTGTCATACTTCTCATGG; human HERV-E: F-GGTGTCACTACTCAATACAC, R-GCAGCCTAGGTCTCTGG; human HERV-F: F-CCTCCAGTCACAACAACTC, R-TATTGAAGAAGGCGGCTGG; human HERV-K: F-ATTGGCAACACCGTATTCTGCT, R-CAGTCAAAATATGGACGGATGGT; human ERV-L: F-ATATCCTGCCTGGATGGGGT, R-GAGCTTCTTAGTCCTCCTGTGT.

### Western blots

Cells were collected in RIPA lysis buffer (Thermo Scientific, cat#89900) containing protease inhibitor mixture (Sigma, cat#P-8340). Protein concentration was quantified using a BCA Protein concentration Kit (Beyotime, cat#P0009). Total cell lysates were subjected to SDS-polyacrylamide gel electrophoresis (PAGE) and transferred to polyvinylidene difluoride membrane. The membrane was incubated with primary antibodies (overnight at 4°C) and sequentially horseradish peroxidase (HRP)-conjugated secondary antibody at room temperature for 1 hour. Membranes were probed with HRP-conjugated secondary mouse antibody (Santa Cruz, cat#sc-516102) at room temperature for 1h. Enhanced chemiluminescence substrates (ECL) (Tanon, cat#180501) were used to visualize the protein abundances. Primary antibodies used were EZH2 (D2C9) (Cell Signaling Technology, cat#5246), TLR3 (TLR3.7) (Santacruz, cat#sc-32232), MDA-5 (D74E4) (Cell Signaling Technology, cat#5321), ISG15 (Cell Signaling Technology, cat#2743), Actin (Santacruz, cat#sc-8432).

### Cell colony formation assay

Cells were trypsinized and resuspended in fresh medium to a single-cell suspension. The suspension was diluted to the desired concentration and seeded into 6-well plates with 500 cells per well or 12-well plates with 200 cells per well. The cells were cultured to grow for 7 days, during which the fresh medium was added on day 4. After removing the medium, the crystal violet solution (0.5% crystal violet powder, 80% H2O, and 20% methanol) was directly added to fix and stain the colony simultaneously. Colony forming areas were quantified by ImageJ software according to the user manual.

### Mouse tumor models

All mouse experiments were approved by The Animal Care and Use Committee of the First Affiliated Hospital of Zhejiang University school of medicine (approved number: 20210422) and conducted according to Institutional Animal Care and Use Committee (IACUC). Female C57BL6 mice at 6-8 weeks old age were purchased from Shanghai SLAC Laboratory animal and maintained under specific-pathogen-free conditions in the animal facility of Zhejiang University. Mice were maintained in temperature-controlled cages under 12-h light-dark cycles with unrestricted food and water availability. Mice were anesthetized with isoflurane (RWD, cat# R510-22), shaved at the injection site, and then subcutaneously inoculated with 0.5x106 LLC tumor cells in 100ul PBS. Tumor sizes were measured using a caliper every 2 days, and the volume of the tumor was calculated as follows: V= (short axis × short axis × long axis)/2. The endpoints were determined by a tumor volume reaching 2000 mm3. For antibody treatment, 100 microgram anti-mouse PD-1 blocking antibody (29F.1A12), (BioXcell, cat# BE0146) and isotype controls (2A3), (BioXcell, BP0089) were injected via intraperitoneal on day 9, day12, day15 post tumor implantation. For lung metastasis assay, mice were injected with 0.2 X 10^6^ B16-F10 (scramble or EZH2 KD) via tail vein. Mice were euthanized and lungs were dissected on day 14 post-intravenous injection. The removed lungs were fixed in Fekete's solution and the visible metastases were counted by two investigators.

### Vector construction and Gene knockdown

For lentivirus production. The pLKO.1-puromycin lentiviral vector or pLKO.1-blasticidin+ lentiviral vectors were used for transducing A549 and LLC cell lines. The shRNA oligo sequences ( for human: sh-Ctrl CCTAAGGTTAAGTCGCCCTCG, sh1-EZH2 CCAACACAAGTCATCCCATTA; sh2-EZH2 CACCGAGAGTACATTATAGGCACCG; sh-TLR3 CCTTACACATACTCAACCT; sh-RIG-1 AATTCATCAGAGATAGTCA; sh-MDA5: CCAACAAAGAAGCAGTGTATA. The sequences for mouse: sh-Ezh2 CGGCTCCTCTAACCATGTTTA; sh-Mda5 CCCATGAGGTATTGTCCTAAA) were annealed and cloned into pLKO.1-puromycin lentiviral vector. Lentivirus with pLKO.1 plasmid was packaged using packing psPAX2 and envelope pMD2.G plasmids in H293T cells at approximately 50% confluence in a complete growth medium. Plasmids were transfected into cells with the help of polyethyleneimine (Invitrogen, cat#BMS1003-1). After 72 h transfection, the virus-containing culture medium supernatant was collected and then passed through a 0.45 mm filter, flowed spinning down at 2000g for 10 minutes to remove debris. To infect cells, collected lentivirus was added to the cells with the medium containing 8μg/ml polybrene. Transduced cells were selected with puromycin at 2μg/ml or blasticidin at 5μg/ml for 5 days before being used and kept expansion with puromycin at 0.5 μg/ml or blasticidin at 1 μg/ml. For double knockdown, the established EZH2 knockdown A549 or LLC cells were then transduced with lentiviral pLKO-sh-MDA5 (or other targets)-Bsd, and selected with both puromycin and blasticidin for 5 days to create double knockdown cell lines.

### TCGA data analyze

The data on EZH2 expression in NSCLC and other types of cancer was from The Cancer Genome Atlas (TCGA) and analyzed by TIMER2.0 (http://timer.cistrome.org/). The Gene_DE module was used to study the differential expression between tumor and adjacent normal tissues for EZH2 across all TCGA tumors. The statistical significance computed by the Wilcoxon test is annotated by the number of stars (* p < 0.05; **p <0.01; *** p <0.001).The prognostic value of EZH2 on survival in NSCLC was analyzed by the Kaplan-Meier plotter (http://kmplot.com). The correlation between EZH2 and infiltration of CD8^+^T in NSCLC was also investigated in TIMER 2.0. The correlation of gene expression was evaluated by Spearman's correlation and statistical significance.

### RNA Sequencing (RNA-Seq) and Analysis

Total purified RNA was isolated from GSK126 or DMSO treated A549 cells using TRIZOL reagent (TAKARA, Cat#9108)) following standard protocols. RNA purity and quantification were assessed using the NanoDrop 2000 spectrophotometer (Thermo Scientific, USA). RNA integrity was evaluated using the Agilent 2100 Bioanalyzer (Agilent Technologies, Santa Clara, CA, USA) and the RNA with RIN > 9 was used to generate libraries. Then the libraries were constructed using TruSeq Stranded mRNA LT Sample Prep Kit (Illumina, San Diego, CA, USA) according to the manufacturer's instructions. The transcriptome sequencing and analysis were performed by OE Biotech Co. Ltd. (Shanghai, China). The RNA sequencing data are deposited at the Gene Expression Omnibus (BioProject accession number: PRJNA831012. https://www.ncbi.nlm.nih.gov/sra/PRJNA831012).

### Statistics analyses

The statistical analyses were performed using R or GraphPad Prism software. All experiments are representative of three independent experiments unless otherwise indicated. Differences between groups were analyzed by two-tailed Students t-test or ANOVA analysis. The significance of statistic was indicated as follows: n.s. not significant, *p< 0.05, **p < 0.01, and ***p < 0.001.

## Result

### EZH2 was elevated in NSCLC and negatively regulate IFNs and APP genes

**Figure 1:**
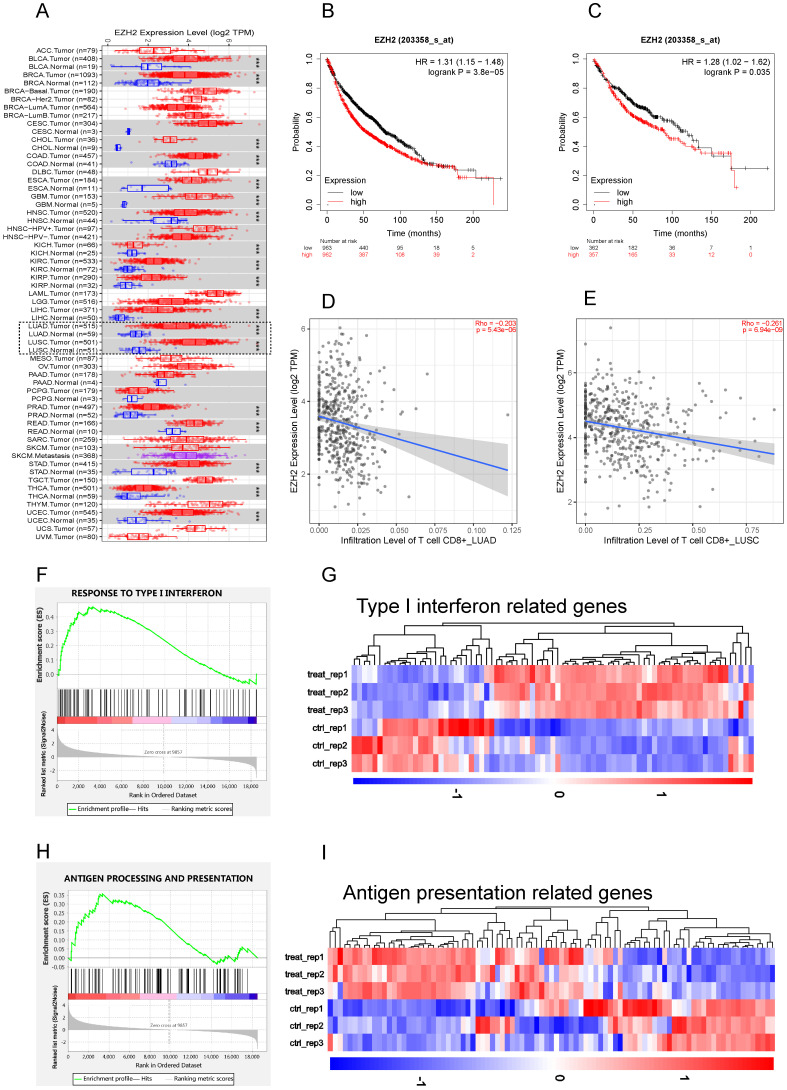
** EZH2 was elevated in NSCLC and negatively regulate IFNs and APP genes.** (A) Human EZH2 expression levels in NSCLC (dotted frame) and other tumor types were analyzed by TIMER 2.0 , The statistical significance computed by the Wilcoxon test is annotated by the number of stars (*p < 0.05, **p <0.01, ***p < 0.001). (B, C) The EZH2 mRNA expression was negatively associated with overall survival in NSCLC (B) and LUAD (C). (D, E) EZH2 expression is negatively related to the infiltration of CD8^+^ T cells in LUAD (D) and LUSC (E). (F, H) GSEA analysis reveals that there is significant upregulation in gene sets response to type I IFN (F, p<0.001) and antigen processing and presentation genes (H, p<0.001) in GSK126 treated A549 cells vs. Control. (G, I) Heatmap for differential expression of type I IFN-related genes (G, FDR <0.05) and antigen processing and presentation related genes (I, FDR <0.05) between control and GSK126 treated A549 cells (gene lists see Supplementary Table 1).

**Figure 2:**
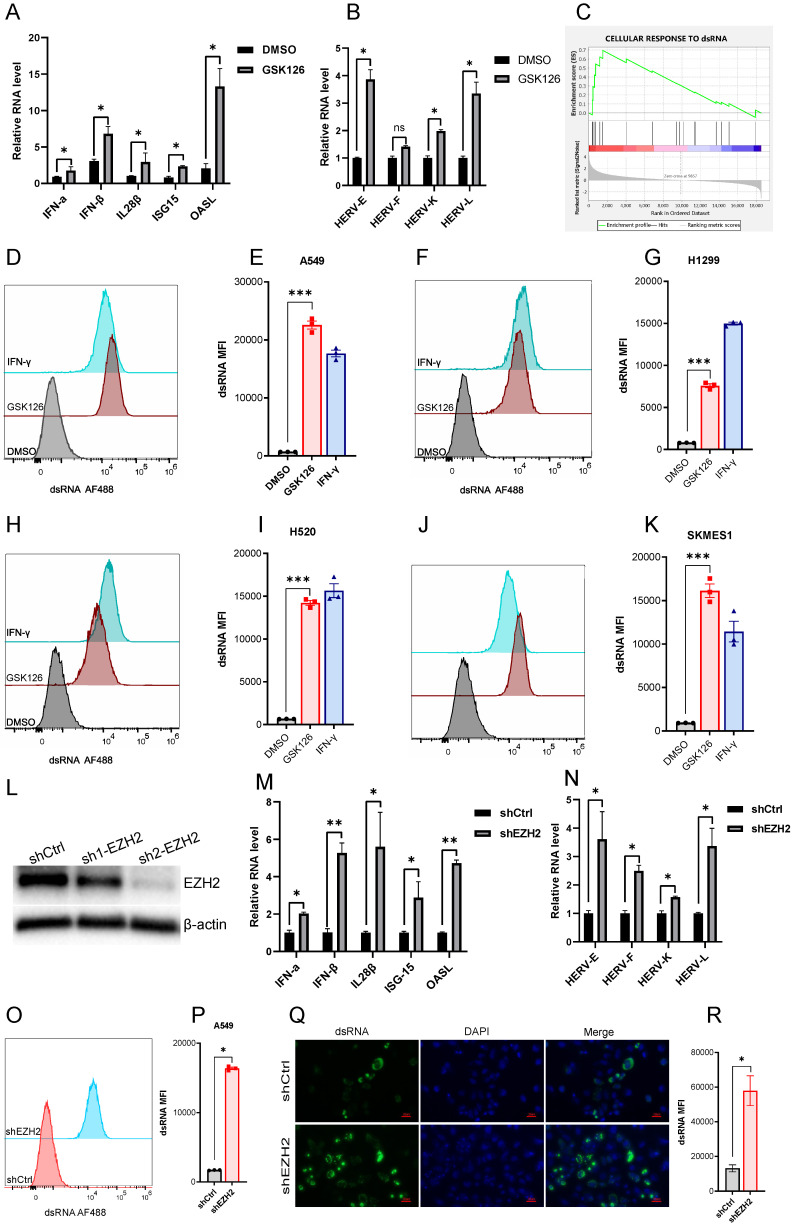
** EZH2 inhibition induces dsRNA expression.** (A) EZH2 inhibition induces the mRNA expression of IFNs and ISGs tested by real time qPCR. (B) EZH2 inhibition induces randomly selected ERVs expression tested by real time qPCR. (C) GSEA analysis reveals that the gene sets response to dsRNA was upregulated in GSK126 treated A549 cells vs. control (FDR<0.05). (D-K) The flow cytometry analysis reveals that inhibition of EZH2 induces the expression of dsRNA in NSCLC cell lines A549(D, E), H1299(F, G), H520(H, I), SKMES1(J, K). IFN-γ as a positive control. (*** indicate p<0.001)**.** (L) Knockdown of EZH2 on protein level was assessed by immunoblotting. (M) The representative IFNs and ISGs mRNA were analyzed by real-time qPCR in EZH2 knockdown A549 cells. (N) The randomly selected ERVs expression was assessed in EZH2 knockdown A549 cells.(O-R) The dsRNA level in EZH2 knockdown A549 cells was tested by flow cytometry (O, P) and immunofluorescence (Q, R). Quantification of dsRNA MFI was followed.

To study the role of EZH2 in antitumor immunity, we first explore the EZH2 expression and its relationship with CD8^+^T cell infiltration on TIMER. According to the TIMER data base, the mRNA expression of 1016 lung tumor tissues and 110 adjacent normal lung tissues were calculated. Compared to parasite tissue, the EZH2 was significantly elevated in NSCLC (Fig 1A). The relationship of EZH2 expression and the overall survival (OS) were explored on Kaplan-Meier Plotter. In this dataset, 1925 NSCLC patients which mainly include 865 adenocarcinoma and 675 squamous cell carcinoma were calculated for OS. The clinicopathological parameters in NSCLC patients were showed in Table 1. The EZH2 expression showed a significant inverse correlation with the overall survival (Fig 1B, C) in NSCLC. The data (Fig 1D, E) imply a negative relation of EZH2 level with the infiltration of CD8^+^T cell in NSCLC. Further, in our RNA sequencing data, the GSEA analysis revealed that inhibition of EZH2 enhances the enrichment of type I IFN (Fig 1F, G) and antigen processing and presentation (APP) gene signatures (Fig 1H, I) in A549 cells. In the differential expression genes analysis, genes related to IFN signaling revealed increases in expression of the APP genes (HLA-A/B/C, B2M, TAP1/2), IFN related genes (STAT1/2, MAVS, ISG15) as well as Th1 chemokines (CCL2, CCL5, and CXCL12). Using the real-time qPCR, we confirmed the RNA-seq data which showed an upregulation of type I and type II IFNs, as well as interferon-stimulated genes (ISGs) (Fig 2A). These results highlight the potential role of EZH2 in regulating immune reactions in NSCLC.

### Inhibiting EZH2 induces dsRNA expression

A prior study demonstrated that derepression of dsRNA by epigenetic target therapy was a key component to trigger the antitumor immune effect 12. The underlying mechanism refers to the 'viral mimicry' that could be induced by the ERVs and other retrotransposons which may contribute to the generation of dsRNAs. To ascertain whether EZH2 inhibition also induced the ERVs, we test a few randomly selected ERVs which were significantly upregulated after EZH2 inhibition (Fig 2B). The GESA analysis revealed the enrichment of genes response to dsRNA in the A549 cells under EZH2 inhibition (Fig 2C). In addition, with the inhibition of EZH2, We test the dsRNA level in some NSCLC cell lines using flow cytometry. The intracellular dsRNA was induced significantly in both lung adenocarcinoma cells and lung squamous cell carcinoma cells (Fig 2D-K). Further, to confirm the EZH2 inhibitor's on-target effect, we knock down the EZH2 in A549 (Fig 2L). The mRNA expression of type I and type III IFNs, ISGs, and prior selected ERVs were increased after EZH2 knockdown (Fig 2M, N). The dsRNA level was also elevated significantly in the EZH2 knockdown A549 cells (Fig 2O, P). Moreover, the dsRNA was assessed by immunofluorescence which further verified the prior flow-cytometry results (Fig 2Q, R). Together, these results suggest that the inhibition of EZH2 induced the elevation of intracellular dsRNA and induced IFN activation in NSCLC. The upregulated ERVs may be part of the source that contributes to the generation of dsRNA.

### EZH2 inactivation stimulating IFN by induction of dsRNA sensors

**Figure 3:**
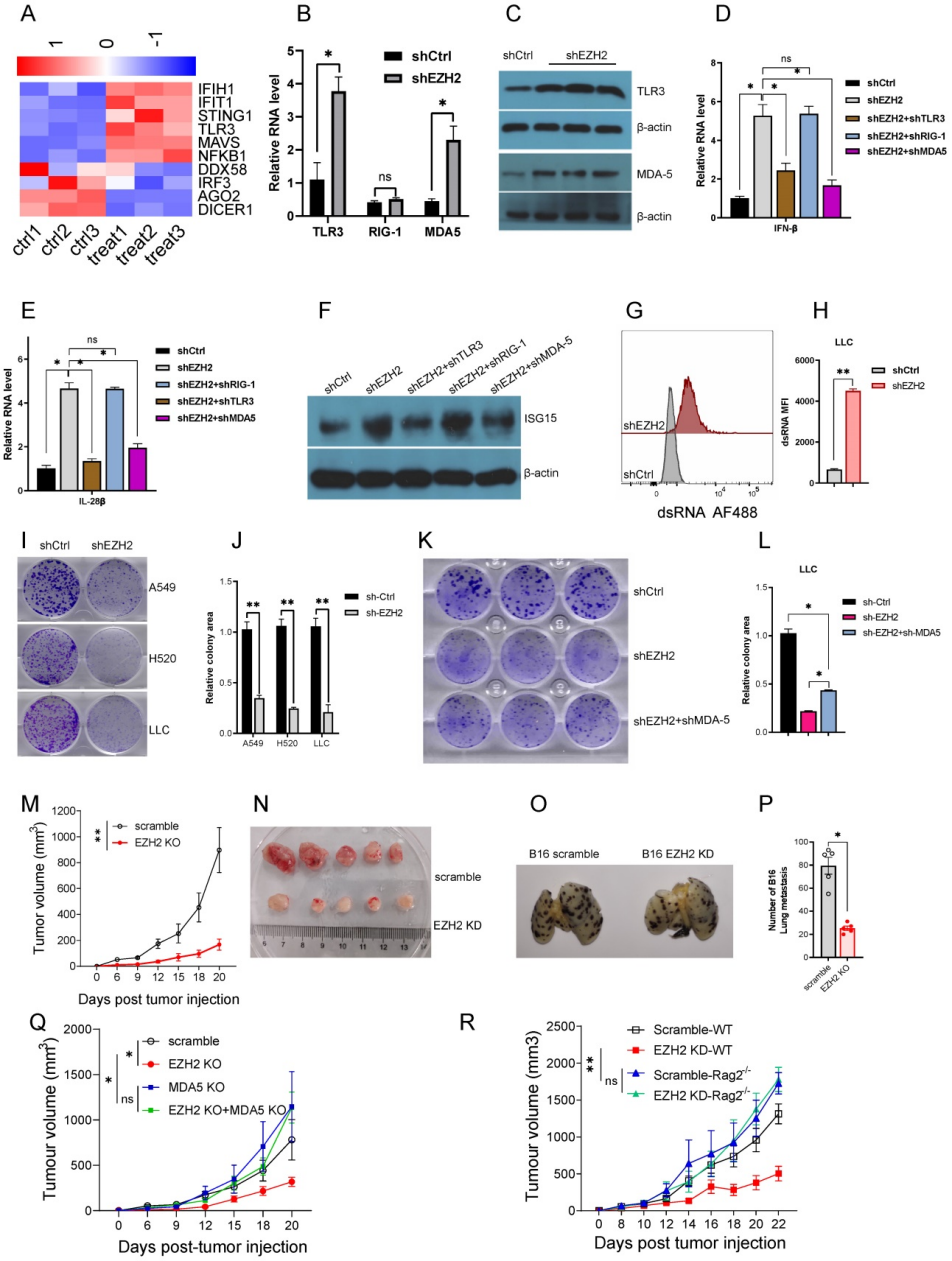
** (A to F) EZH2 inhibition causes dsRNA sensor upregulation and triggers IFNs activation. (G to R) EZH2 abrogation inhibits tumor growth both *in vitro* and *in vivo*.** (A) Heatmaps for differential expression of gene response to dsRNA (FDR<0.05).(B) Pattern recognition receptors, TLR3, RIG-I, and MDA5, were analyzed by real-time qPCR on mRNA level. (C) TLR3 and MDA5 protein levels were tested by immunoblotting in EZH2 knockdown A549 cells. (D, E) Real-time qPCR analysis of IFNβ (D) and IL-28β (E) upon knockdown with targeting EZH2 alone or combining with pattern recognition receptors.(F) Immunoblotting analysis of ISG15 expression when knockdown with indicated shRNA in A549 cells.(G, H) EZH2 knockdown induces dsRNA expression in LLC cells (G), and dsRNA MFI followed (H). (I, J) Colony formation in NSLCL and LLC cells with EZH2 scramble or knockdown was quantified.(K, L) Colony formation in LLC with scramble, EZH2 knockdown, or EZH2 and MDA5 double knockdown was quantified. (M) Average tumor growth curves of subcutaneously inoculated with scramble or EZH2 KD LLC cells in C57BL6 mice. (N) Representative images of tumors in C57BL6 mice from the scramble group and the EZH2 KD group. (O, P) Representative lung metastasis images (O) and quantification (P) of immunocompetent mice receiving scramble or EZH2 KD B16 cells intravenously. (Q) Average tumor growth curves of subcutaneously inoculated with scramble or EZH2 KD, or EZH2 and MDA5 double KD of LLC cells in C57BL6 mice. (R) Tumor growth of immunocompetent (WT) or immunodeficient (Rag2^ -/-^) mice injected with scramble or EZH2 knockdown LLC cells. One-way ANOVA or two-tailed Student's t-test was performed for statistical analysis; *P < 0.05, **P < 0.01.

The intracellular dsRNA applying a “viral mimicry” machinery is recognized by its sensor named pattern recognition receptors, TLR3, MDA5, and RIG-1, which are subsequently involved to activate IFN pathways. In the RNA-seq data, EZH2 inhibition cause the elevation of TLR3 and IFIH1 (encoding MDA5) but not DDX58 (encoding RIG-1) (Fig 3A). The MAVS is the downstream adaptor of the MDA5 pathway which was also induced by EZH2 inhibition. The TLR3 and MDA5 elevation in both mRNA and protein levels was confirmed in EZH2 knockdown A549 cells (Fig 3B, C). In addition, to distinguish which sensor is crucial for IFN activation, we performed double knockdown of EZH2 and dsRNA sensors. The IFNβ and IL-28 as well as ISGs could be significantly rescued by knockdown of TLR3 or MDA5 but not RIG-1 (Fig 3D, E, F). Therefore, these results indicate that dsRNA recognition by TLR3 and MDA5 is crucial for IFN activation when EZH2 inhibition.

### EZH2 inactivation inhibits lung tumor cell growth both *in vitro* and *in vivo*

To investigate the biological effect of EZH2 inhibition, we first test whether the dsRNA can be induced in mouse Lewis lung carcinoma (LLC). As expected, EZH2 knockdown induced upregulation of dsRNA in LLC (Fig 3G, H). The colony formation data showed that EZH2 knockdown results in compromised cell growth both in NSCLC cells and LLC cells (Fig 3I, J). To determine that the cell growth inhibition is dependent on EZH2 inhibition induced dsRNA stress, we knockdown MDA5, and EZH2 concurrently in LLC. The inhibition of MDA5 partially rescued the cell growth suppression incited by EZH2 inhibition (Fig 3K, L). These results suggest that the EZH2 knockdown induced dsRNA stress is partially responsible for the tumor growth defect.

To investigate the *in vivo* effect of EZH2 inhibition, the LLC was subcutaneous injected into the immunocompetent C57BL/6 mice. Compared with the scramble group, the EZH2 knockdown group tumor growth was significantly inhibited (Fig 3M, N). The B16 tumor cells were used to assess the lung metastasis, in the EZH2 knockdown cells, the lung metastasis were reduced significantly (Fig 3O, P). In addition, to determine that the antitumor effect was induced by dsRNA stress, the EZH2 knockdown, and MDA-5/EZH2 double knockdown LLC was implanted on C57BL6 mice. Inconsistent with the *in vitro* results, the tumor regression caused by EZH2 knockdown was partially rescued by MDA-5 inhibition (Fig 3Q). Next, we wanted to distinguish the role of EZH2 on tumor cell autonomous effect versus adaptive anti-tumor immunity. The LLC cells were injected subcutaneously into the immunocompetent C57BL6 and immunodeficient Rag2^ -/-^ mice. As expected, in the Rag2^ -/-^ mice, the tumor volume had no significant difference between EZH2 inhibition and the control group (Fig 3R). These data imply that EZH2 inhibition causes LLC regression through eliciting endogenous anti-tumor immunity, rather than affecting tumor cell autonomous growth.

**Figure 4:**
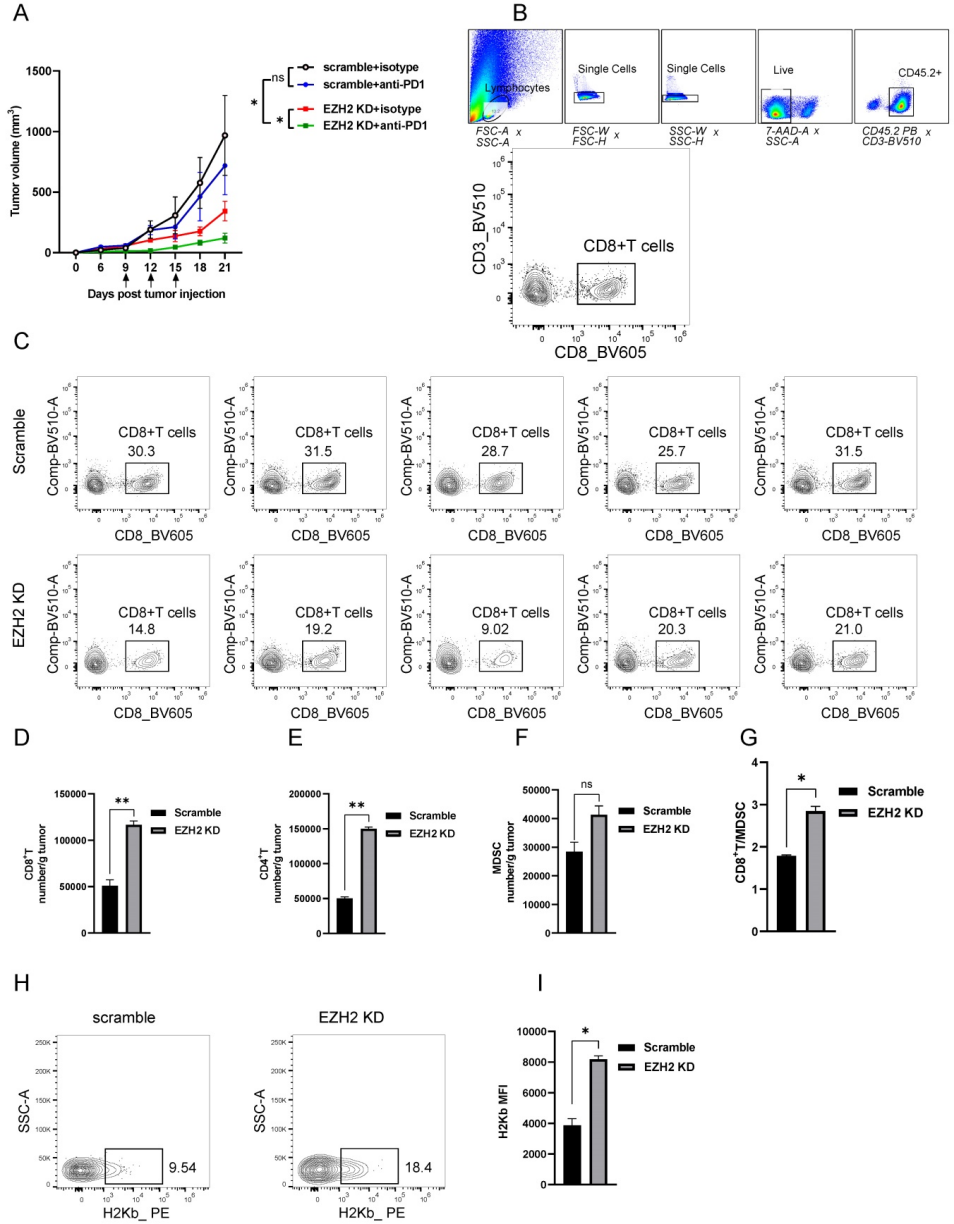
** EZH2 Inhibition enhances lung tumor immunogenicity.** (A) Average tumor growth curves of C57BL6 mice inoculated with LLC cells and treated with anti-PD-1 or isotype control. Arrows indicate time points of 100ug/mouse anti-PD-1 injection. (B, C) The tumor infiltration lymphocytes gate strategy (B) and representative dot plot of CD8^+^T cells (C) were shown. (D to G) Tumor infiltrating lymphocytes were analyzed by flow cytometry from LLC tumors (scramble n=5, EZH2 KD n=5), the number/gram of CD8^+^T (D), CD4^+^T (E), MDSC (F), and the CD8^+^T/MDSC ratio (G) was shown. (H, I) MHC-I level of LLC tumor isolated from C57BL6 mice was analyzed by flow cytometry, representative dot plot (H), and the MFI was followed. Unpaired t-test was used for statistical analysis. Images are representative of two biological replicates. MFI error bar presents as mean ± SD. *p < 0.05, **p < 0.01, ns, not significant.

Furthermore, to explore whether the EZH2 inhibition has a synergistic effect with checkpoint blockade therapy, we injected the anti-mouse PD1 antibody into the EZH2 knockdown tumor model. Compared with the scramble group, PD1 blocked alone had no superior tumor regression (Fig 4A). The group of EZH2 knockdown combined with PD1 blocker had significant tumor size regression (Fig 4A). Therefore, these results indicate that the dsRNA stress induced by EZH2 inhibition facilitates tumor compromise. The EZH2 knockdown could enhance tumor response to immune checkpoint blockade therapy.

### Inhibition of EZH2 promotes T Cell Infiltration and Enhances Tumor Immunogenicity

To reveal the mechanism of EZH2 inhibition caused tumor regression *in vivo*, we analyzed the immune cell feature in the tumor microenvironment. The detailed gate strategy of tumor infiltration lymphocytes representative dot plot of endogenous CD8^+^T cells was shown (Fig 4B, C). Compared with the scramble group, the EZH2 knockdown LLC tumors had significantly increased infiltration of CD8^+^T and CD4^+^T cell numbers (Fig 4D, E). In addition, the CD8^+^T/MDSC (Myeloid-derived suppressor cells) was significantly increased in the EZH2 inhibition group, in spite of the MDSC cell number having a slight increase (Fig 4F, G). To explore the role of EZH2 inhibition in regulating antigen presentation of tumor cells *in vivo*, we analyzed the tumor samples after tumor implantation for 12 days. In the EZH2 knockdown group, tumor cell MHC class expression levels were increased compared with the control group (Fig 4H, I). These results demonstrate that EZH2 inhibition promotes T cell infiltration and enhances tumor immunogenicity.

## Discussion

In the present study, we have found that EZH2 inactivation induced the expression of ERVs and triggers dsRNA stress which leads to type I and types III IFN responses in NSCLC. In the mouse model, we demonstrated that EHZ2 inhibition caused tumor regression and had a sensitization effect on PD1 blockade. Further analysis showed that EHZ2 inhibition promotes the recruitment of immune cells to tumors and the tumor expression of antigen presentation genes. Our study provides evidence that supports EZH2 as a negative epigenetic regulator of antitumor immunity and responsiveness to ICIS therapy. These data suggest EZH2 inhibition may as a means to enhance NSCLC response to anti-PD1 therapy.

In recent years, accumulating evidence suggests that epigenetic regulation was involved in tumors to evade immune eradication and resistance to immunotherapies 12, 14, 17. The dsRNA was a major element to elicit immunogenicity and triggers immune response both in virus infection and tumor microenvironment. Our data showed that inhibition of EZH2 induces the expression of dsRNA and the transcription of a subset of ERVs in NSCLC cancer cells. In our RNA-seq data, we found that EZH2 inhibition leads to a reduction of the dsRNA cleaving enzyme, DICER (Fig 3A), which may explain how this regulation can contribute to the elevated level of dsRNA in EZH2 knockdown cells. EZH2 inhibition also results in a reduction of AGO2, which may cleave complementary RNA transcripts that form dsRNA, thus reducing dsRNA formation 18. Above all, EZH2 inhibition could cause dsRNA stress and subsequent immune responses possibly by regulating ERVs transcription and dsRNA cleaving related enzyme expression. We also found that EZH2 inhibition can elicit intracellular dsRNA stress and resultant cellular type I and type III IFN activation. During the activation of the IFN pathway, the intracellular dsRNA is recognized by pattern recognition receptors, including MDA5, TLR3, and RIG-I 19. Our results demonstrated that MDA5 and TLR3 are crucial for recognizing dsRNA to IFNs activation. These results are consistent with a recent study that shows LSD1 inhibition trigger dsRNA sensor recognition depending on MDA5 and TLR3 but not RIG-I in a breast cancer cell line MCF-7 12. This study also proved that STING as a sensor of cytoplasmic DNA is unlikely the trigger of IFN responses. In another recent study, the EZH2 inhibition could stimulate IFNs through the activation of dsRNA in prostate cancer. Interestingly, they found crosstalk of EZH2 and STING activity and suggest that the activation of IFN-stimulated molecules is partially dependent on STING activity 14. In our data, EZH2 inhibition also induced STING1 expression (Fig 3A), but whether activation of STING is could sense dsRNA stress is another interesting topic that should be further investigated in NSCLC.

In the present study, we found that EZH2 KD could reduce the NSCLC cell growth *in vitro*. However, when the EZH2 KD LLC were implanted in Rag2^-/-^ mice, the growth between WT and EZH2 KD tumors had no significant difference. This inconsistent phenomenon also was reported in Sheng et al's study 12, in which they found LSD1 KO does inhibit the growth *in vitro* but not in TCR-a KO mice. These interesting issues suggest that epigenetic regulators (like LSD1 or EZH2) regulate tumor cell growth *in vitro* through both dsRNA-IFN stimulation and possibly an intrinsic proliferation program. We speculate that in the tumor microenvironment, the host somatic cells (like stromal cells) could foster cell proliferation that may circumvent the effect of EZH2 on cell proliferation *in vivo*. Another possible speculation is that the innate immune pressure acting on tumor cells minimizes the growth difference between WT and EZH2 KD tumors. Above all, this is an issue had not been fully clarified in our and other studies and needs to be further studied.

With the emerging therapy of ICIs but low response rate, identifying mechanisms driving resistance to anti-PD1/PDL1 in NSCLC patients remain a critical requirement. Our results demonstrated that targeting EZH2 therapy reprograms the tumor immunogenicity by inducing a significant increase in CD4^+^ and CD8^+^ T cells within the tumor microenvironment. As a key antigen presentation component, the MHC-1 elevation in the tumor also supports the lymphocytes infiltration and immune therapy. Indeed, inconsistent with our data, EZH2 inhibition had shown a synergistic effect with anti-PD1 therapy in prostate and melanoma cancers 14, 20. Consistent with our findings in LLC models, the TCGA data reveals a negative correlation between EZH2 level and CD8^+^ T cell infiltration in NSCLC patients. These data suggest a therapeutic potential of targeting EZH2 in combination with anti-PD1/PDL1 for NSCLC treatment. Moreover, the TCGA data reveal that the EZH2 expression was elevated in a variety of cancers and associated with poor prognosis which further suggests EZH2 expression may be a significant biomarker in NSCLC.

## Conclusion

In summary, the present study revealed that epigenetic mechanisms mediated by EZH2 inhibition induce dsRNA intracellular stress, resulting in an increased type I and type III IFN response within tumor cells, thereby altering the tumor microenvironment and enhancing the tumor response to PD1 blockade. These findings generate the rationale that targeting EZH2 in combination with anti-PD1 /PDL1 may be an applicable strategy in NSCLC therapy.

## Supplementary Material

Supplementary table.

## Figures and Tables

**Table 1:** The clinicopathological parameters in NSCLC patients for the OS analysis

Characteristics	EZH2 expression			
	Low	High	χ^2^	*P* value
Gender				
Female	357	357	<0.001	>0.99
Male	550	550		
Smoke				
Yes	414	406	0.04	0.85
No	102	103		
Histology				
Adenocarcinoma	362	357	0.003	0.96
Squamous cell carcinoma	263	261		
TNM Stage				
T				
1	218	219	0.045	0.97
2	297	292		
3+4	40	41		
N				
0	391	390	<0.001	0.98
1+2	182	181		
M				
0	340	341	0.09	0.76
1	6	5		
Grade				
I	100	101	0.03	0.98
II	156	154		
III	38	39		

## Abbreviations

NSCLC: non-small cell line cancer
EZH2: Enhancer of Zeste 2 Polycomb Repressive Complex 2
dsRNA: double-strand RNA
TLR3: Toll-Like Receptor 3
MDA5: melanoma differentiation-associated protein 5
LLC: Lewis lung carcinoma cells
PD1: Programmed Cell Death 1
PDL1: Programmed death-ligand 1
ICIS: immune checkpoint inhibitors
IFNs: interferons
ERVs: endogenous retroviruses
DNMT1: DNA Methyltransferase 1
